# Risk factors for child abuse: levels of knowledge and difficulties in family medicine. A mixed method study

**DOI:** 10.1186/s13104-015-1607-9

**Published:** 2015-10-30

**Authors:** Océane Regnaut, Marie Jeu-Steenhouwer, Cécile Manaouil, Maxime Gignon

**Affiliations:** Medical School, University of PicardyJules Verne, 3 Rue des Louvels, 80000 Amiens, France; Forensic and Social Medicine Unit, Amiens University Medical Center, 80054 Amiens Cedex 1, France; Primary Care Department, Jules Verne University of Picardy, 3 Rue des Louvels, 80000 Amiens, France; Laboratory Educations and Health Practices, EA 3412, University Paris 13, 74, Rue Marcel Cachin, 93017 Bobigny, France; Health Training Center (SimUSanté©), Amiens University Medical Center, 80054 Amiens Cedex 1, France

**Keywords:** Primary care, Family physician, Child abuse, Detection, Public health

## Abstract

**Background:**

Family physicians (FPs) have a central role in the detection and management of child abuse. According to the literature, only 2–5 % of initial reports of child abuse come from the medical profession.

**Methods:**

The objective of this study was to assess levels of knowledge of risk factors for child abuse by Family Physicians (FPs) and the attention that the physicians pay to these risk factors. We conducted a mixed-method survey based on semi-structured interviews. 50 FPs practicing in the Somme County (northern France) were interviewed with closed and open questions. The FPs’ level of knowledge of risk factors for child abuse and obstacles in the detection of child abuse were assessed.

**Results:**

The FPs’ level of knowledge of risk factors for child abuse was similar to that reported in the literature. However, FPs knew little about the significant role of prematurity. Likewise, the FP’s training did not seem to influence their knowledge of risk factors. Fear of an incorrect diagnosis was the main obstacle to reporting a suspected case. The FPs considered that they were often alone in dealing with a difficult situation and considered that the judicial system and the social services were not sufficiently active.

**Conclusions:**

Few FPs had actually received specific training in the detection and management of child abuse but many stated their need for this type of training. FPs encounter many obstacles in the detection of child abuse, which sometimes make the FP reluctant to report a suspected or potential case. Medical education need to be improved in this field.

**Electronic supplementary material:**

The online version of this article (doi:10.1186/s13104-015-1607-9) contains supplementary material, which is available to authorized users.

## Background

The family physician (FP) has a key role in detecting and caring for abused children or those at risk of being abused. A suspected case of abuse is always difficult to deal with and some physicians will be unsure of how to act [1].

France has well codified systems for dealing with suspected or potential child abuse (Additional file 1) [2]. In France, if a FP suspects abuse, he must do everything possible to protect the child. It may report to the District Prosecutor in writing or by phone with subsequent written confirmation. The District Prosecutor begins a criminal investigation and, if necessary, also contacts the county social services. If a FP identified risks of abuse (deficient care or education), he can contact the county child abuse prevention office. This office starts to evaluate the child’s situation. If necessary, the office she sends the file to the District Prosecutor with a view to legal proceedings.

However, only 2–5 % of reports come from the medical profession [3, 4]. Most reports are made by social services, schools and hospitals.

Several studies have sought to identify the difficulties faced by FPs and that might explain this “under-reporting”: fear of an incorrect diagnosis [5, 6], the possible impact of a report on the FP’s relationship with the family [6], fear of not spending enough time with other patients and, lastly, financial repercussions [5–7]. In study by Jones’ et al. [8], 83 % of the physicians explained that they had failed to report an incident because they knew the parents well.

Other research has found that physicians are less inclined to make a report because they feel that this action will not benefit the child, [7–9] and may even aggravate the situation. [6, 10] Physicians also mention a lack of confidence; [5, 6, 11] or a previous negative experience with social services or the judicial system [8, 10, 12, 13]. They are also afraid of the penal repercussions for FP and/or parents, and considered that making and following up a report is very time-consuming [11].

Prevent child abuse and improve screening should help overcome these obstacles. However, for this type of approach to be effective, FPs must be aware of the risk factors for child abuse. Certain researchers have insisted on the need for physicians to learn to recognize these risk factors and thus handle difficult situations more easily [14]. Risk factors for child abuse are those likely to weaken the child’s relationship with her parents; some are well established and others are still subject to debate (Additional file 2).

The present study’s primary objective was to estimate FPs’ level of knowledge of the risk factors for child abuse. The study’s secondary objectives were to estimate FPs’ use of this knowledge to identify suspected or potential abuse in their practices, identify obstacles to dealing effectively with suspected child abuse, gauge the FPs’ feelings about their role in managing child abuse, determine the type and level of any specific training received and estimate the FPs’ stated requirements for training.

## Methods

The study was based on 50 interviews with FPs in private practice in the Somme County (northern France). We used a hetero-administered questionnaire with open and Likert scale questions questions during interview (Additional file 3).The interviews were performed by one interviewer between July 2011 and May 2012.

### The study population

The study population was drawn from the list of the FPs in private practice in the Somme on July 1st, 2011, according to the Picardy Association of Private Practice Physicians. A total of 577 FPs were included in the study selection database.

We arbitrarily set a target sample size of 50 FPs. On the basis of a participation rate of 25 % observed in similar methodological studies, 200 FPs were selected at random from the database by using HAZARD^®^ software. The selected FPs were contacted sequentially by telephone until 50 had agreed to participate in the study.

### Data collection

Each individual interview in the FP’s office was based on a set of 30 closed or open questions and lasted for between 15 and 30 min. The first part of the interview focused on the FP’s sociodemographic characteristics and the second part looked at how the FP would deal with suspected child abuse and probed their knowledge of the risk factors. The third part questioned the FP about their perceived role in detecting and managing suspected child abuse and their opinion of the other stakeholders in the area of child abuse. The fourth and last part of the interview focused on the FP’s past training (if any) and perceived training needs. The interviews were conducted in French and were transcribed and analysed by native French speakers. This study was approved by the Committee of Primary Care of the university. FP were informed and all participants provided their informed consent.

### Analysis

A descriptive analysis of these data was performed using SPSS software (version 11.0, SPSS, Inc). Quantitative variables were expressed as the mean ± standard deviation and qualitative variables were expressed as the frequency and percentage.

In a second step, a bivariate analysis of the data was performed by using the Chi squared test or the Fisher’s exact test. The threshold for statistical significance was set to *p* < 0.05. A multivariate analysis could not be validly applied because of the small sample size.

In a third step, answers to open-ended questions were analyzed with a qualitative approach. Answers related to a common concept were then gathered together. Open responses were analyzed qualitatively, in order to identify the concepts developed. The transcripts were entered into Tropes^®^ for content analysis and then coded according to the session headings. After content analysis, the discussions were recoded according to the themes that had emerged. Lastly, the answers were grouped into categories, in order to summarize the opinions.

## Results

### Disposition of the study group

To achieve the target of 50 physicians, we called 173 FPs so the response rate was 28.9 %. The median age was 52 and 38 of the FPs (76 %) were men. All FP are born and graduates in France. The FPs mainly practiced alone (n = 21, 42 %) in semirural areas (n = 22, 44 %). The others FPs practiced in rural areas (n = 16, 32 %) or urban areas (n = 12, 24 %).

### Perception of the risk factors by the FPs

Ninety percent of the FPs considered that there are risk factors for child abuse, whereas 3 (6 %) considered that there are none at all. All the results are summarized in Table 1.

**Table 1:** Perception by general practitioners of risk factors for child abuse

	Strongly disagree n (%)	Tend to disagree n (%)	Tend to agree n (%)	Strongly agree n (%)	No answer n (%)
Risk factors for child abuse related to the child					
Young age	19 (38 %)	4 (8 %)	10 (20 %)	16 (32 %)	1 (2 %)
Prematurity	36 (72 %)	7 (14 %)	5 (10 %)	0	2 (4 %)
Mental handicap	6 (12 %)	5 (10 %)	21 (42 %)	18 (36 %)	0
Physical handicap	9 (18 %)	8 (16 %)	19 (38 %)	13 (26 %)	1 (2 %)
Behavioral disorders	2 (4 %)	2 (4 %)	23 (46 %)	22 (44 %)	1 (2 %)
Risk factors for child abuse related to the parents					
Emotional deficiency	4 (8 %)	4 (8 %)	16 (32 %)	25 (50 %)	1 (2 %)
Failure to provide parental care	8 (16 %)	12 (24 %)	14 (28 %)	16 (32 %)	0
Psychiatric disorders	5 (10 %)	2 (4 %)	13 (26 %)	26 (52 %)	4 (8 %)
Depression (especially post-partum)	4 (8 %)	4 (8 %)	20 (40 %)	19 (38 %)	3 (6 %)
Drug and/or alcohol abuse	0	0	2 (4 %)	48 (96 %)	0
History of abuse in a parent	2 (4 %)	4 (8 %)	13 (26 %)	29 (58 %)	2 (4 %)
Parental inability to meet the child’s requirements	2 (4 %)	1 (2 %)	20 (40 %)	26 (56 %)	1 (2 %)
Young maternal age	16 (32 %)	8 (16 %)	14 (28 %)	10 (20 %)	2 (4 %)
Low maternal educational level	6 (12 %)	9 (18 %)	18 (36 %)	16 (32 %)	1 (2 %)
Disadvantaged socio-economic status	3 (6 %)	5 (10 %)	16 (32 %)	25 (50 %)	1 (2 %)

Some doctors talked about their desire for screening tools: “I will use it to simplify the screening, I like what we can quantify. A scale is reassuring official” or “Just to have this tool available is relevant. If we have the tool, we think more about screening, thus increasing our experience in this field and we are more vigilant”. Others are more reserved, “it’s not a scale that will tell me if a child is being abused.”

### The physicians’ own experience

Eighty-two percent of the FPs (n = 41) had already suspected a case of child abuse and had identified risk factors beforehand in more than half of these instances. Of these FPs, 31 (62 %) stated that their diagnosis had been strengthened by the presence of risk factors. In most cases, the child’s consultation had been followed by hospitalization. Ten of the FPs had filed an administrative report and 12 (25 %) had contacted the State Prosecutor. Some of the FPs preferred to ask the social services for advice, to discuss the case with a colleague or investigate matters further themselves.

In four cases, the report was not followed by legal proceedings. The child abuse was confirmed in 61 % of cases (n = 25); 64 % (n = 16) in the presence of risk factors and 50 % (n = 6) in the absence (*p* = 0.7).

Only nine FPs (18 %) had never encountered a case of suspected or potential child abuse. Fifty-six percent considered that the presence of risk factors would not strengthening their diagnosis. More than half declare to have no barriers in the support of a case of child abuse.

### Difficulties encountered in cases of suspected child abuse

**Fig. 1:**
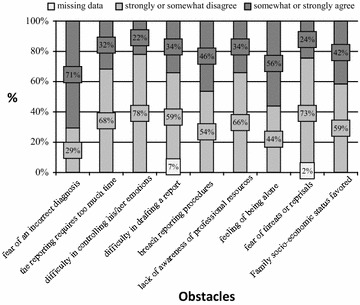
The obstacles encountered by the family practitioner when faced with suspected child abuse

The most frequently mentioned problem was the difficulty in establishing a diagnosis of child abuse (Fig. 1). Almost all the FPs stated that protecting the child was the major driver for their actions. Diagnostic certainty and being able to count on help from third parties were also motivating factors.

The impact of a procedure for the child and his family are a hindrance, that reporting is done wrongly or rightly, “I may explode the family”; “I’m afraid of losing sight of the child”; “The risk is that the report due to bringing more harm to the child that if I did not report.”

Many doctors criticize relations with social services, seeing them as being unresponsive, holding that the monitoring of the child they propose is not always consistent, “they show considerable inertia”, “workers social are useless," “social services? Either they panic for nothing, or they are missing out on real life situations".

### The physicians’ feelings about the role in caring for potential victims of child abuse

Most of the FPs (n = 47, 94 %) stated that they felt personally involved in caring for children at risk of child abuse. However, once child abuse had been identified, less than about ten of them refer the child and her family, even report.

Many of the FPs (n = 39, 78 %) did not feel that they were sufficiently involved in the process by the other stakeholders in the field of child abuse (pediatricians, school or forensic physicians). Some of the surveyed FPs (n = 10, 20 %) deplored the lack of information about the consequences of reporting suspected child abuse and sometimes went as far as criticizing the action of the social services (e.g. lack of responsiveness, poorly coordinated follow-up, etc.).

Some doctors emphasize their personal and professional conscience, “I will act so as not to be angry with me all my life if I close my eyes to a situation of child abuse.”

### Past and future training

Only 15 of the FPs (30 %) had received training on detecting and managing child abuse and over half of these had found it useful. Of those who had not received any training, 35 (78 %) wished to receive training in the future; the remainder considered that they did not need any additional training.

The FPs stated that the training course should be compact, practice-based, FP-led and up-to-date (to reflect changes in the legislation) and should be delivered in a university-level institution. More than half of the FPs (n = 27, 54 %), suggested that a 24/7 medical support line would be useful.

We did not observe significant differences between FPs having received specific training and those not having specific training in terms of knowledge of risk factors for child abuse. However, there was a statistically significant difference between trained and untrained FPs in terms of their level of knowledge of the reporting procedures (71 *vs* 33 %, p < 0.05).

## Discussion

### Knowledge of risk factors and perception of the value of these factors in clinical practice

Forty-five of the 50 FPs (90 %) in our study considered that there are a number of risk factors for child abuse. The FPs were not greatly aware of the key role of prematurity and psychological and emotional factors; they considered that disadvantaged socioeconomic status was more important. It is probably more difficult, for the practitioners, to detect the child abuse in the favored environments (higher socioeconomic status), especially as they belong to them. The French Social Action Observatory has emphasized that psychological and emotional factors are more important that low socioeconomic status in the risk of child abuse [15]. This was confirmed by Tursz et al.’s study, in which most of the mothers having shaken their child had an higher socioeconomic status [16].

According to the literature, there are many risk factors for child abuse (Additional file 2). However, low educational level and disadvantaged socioeconomic status are subject to debate. [15–19] According to Flaherty et al., the level of suspicion of child abuse should be higher when the practitioner identifies the presence of a risk factor. [20].

### Difficulties encountered in cases of suspected child abuse

The main difficulty evoked by the FPs of our study was the fear of making an incorrect diagnosis. In the literature, the fear of making a mistake is also the main obstacle to reporting (along with the judicial consequences for the practitioner) [5, 7, 13].

### Roles in reporting child abuse

Even in clear cases of abuse, physicians fear that breaking up the family unit will have even more harmful consequences for the child. This aspect is also related to the widely reported feeling that judicial and social services are not sufficiently active or do not offer adequate solutions. [5–8, 13, 21] Some FPs report a negative experience with social services or judicial, for the child or for themselves, which does not encourage them to report or to screen. This difficulty is compounded by the fact that 46 % of the FPs having suspected child abuse had little or no knowledge of the reporting procedures. Poor understanding reporting procedures was also cited as a possible obstacle by nine FPs who had not been confronted with a suspected case of child abuse [9].

### Training now and in the future

Training is one possible solution. However, although this is a legal obligation in France, few FPs stated that they had received specific training. An important aspect in line with the literature is the fact that most professionals did not attend training on the thematic of violence against children and adolescents. Other authors highlight the scarce knowledge about the issue as one of the main factors affecting the identification and reporting of maltreatment [22, 23]. An other study undertaken with professionals of Primary Health Care in Northern Ireland found that many workers failed to report for not knowing how to proceed in cases of abuse. [6].

There was a significant difference between FPs having received training and those who had not; the former were more likely to state that that did not understanding the reporting procedure. This may be because training increases the physician’s awareness of the complexity of the situation without helping him/her in practical terms.

### FPs do not have a particular focus

One can hypothesize that FPs do not have a particular focus child abuse during their medical training because they are rarely confronted with this problem in practice. Even though training on detecting and managing child abuse is a part of the French national curriculum for sixth-year medical students, it is not a greatly studied topic.

Other tools mentioned by the FPs included a national hotline for physicians. A French national hotline for reports of suspected abuse (including reports by children themselves) already exists, and county child protection units often offer similar services? However, these hotlines are not specifically targeted at and staffed by physicians (an idea also suggested by Flaherty et al.) [12].

### Study limitations

Recruitment bias was a potential limitation; since participation was voluntary, practitioners who were more interested in and comfortable with the subject were probably more like to agree to participate. A third potential source of bias related to the vocabulary used in the study; before starting the interview, we did not remind the FP of the main definitions in this field (emotional deprivation and the parents’ inability to meet the child’s needs).

### Implications for clinical practice and research

Overall, our findings are in agreement with the literature data on risk factors for child abuse. Nevertheless, they did not know much about the predominant role of prematurity and psychological/emotional factors (as opposed to socioeconomic factors) in the genesis of child abuse. Insisting on this point in specific trainings for FPs might increase the detection of suspected child abuse. With regards to the current legal obligation, it would be advisable to extend this training to all FPs and to make it very practically focused and rich in case studies.

In contrast to several larger studies performed outside France, our survey of the obstacles to reporting did not highlight a particular interest in this type of training [7, 10, 20, 24].

One of the major obstacles mentioned by the FPs in our study related to the impact of the report on the child and its family if child abuse was confirmed. We did not assess the FPs’ knowledge of the various types of support available to the child and its family following a report. It may be of value to provide FPs with more information about these measures and to show that the overall outcome of support is beneficial for the families (as suggested by Flaherty et al. in 2000) [7].

Similarly, changing the FP’s view of the social and judicial services appears to be essential if practitioners are to improve their detection and management of suspected child abuse. A Canadian survey of pediatricians suggested informing them more precisely about the roles of the social services and their own difficulties when faced with an at-risk child [25]. It may be essential to increase the feedbacks of the social and judicial services and of the system to the FP, giving the feeling to the latest to belong to a multidisciplinary team and that their action is not useless [7, 8, 10].

Along with a well-designed training program, the surveyed FPs suggested that a support hotline would be of great value. Working as part of a network with specialist hospital units may be a source of support for primary care physicians [24].

## Conclusions

There are many obstacles in FP’s to perform their role in protecting children from abuse. It is important to offer the FP support (in terms of the availability of training and multidisciplinary networks) so that the physician does not have to deal with these challenging situations alone.

## Additional files

10.1186/s13104-015-1607-9 Courses of action in a suspected case of actual or potential child abuse in France.
10.1186/s13104-015-1607-9 Risk factors for child abuse reported in the literature.
10.1186/s13104-015-1607-9 Questionnaire.
