# Supplementary material for: Risk factors for child abuse: levels of knowledge and difficulties in family medicine. A mixed method study
Source: BMC Res Notes. 2015 Oct 30;8:620. doi: 10.1186/s13104-015-1607-9 (PMC4627620; doi:10.1186/s13104-015-1607-9)
Supplement: Supplementary file 1 — 10.1186/s13104-015-1607-9 Courses of action in a suspected case of actual or potential child abuse in France. [file 13104_2015_1607_MOESM1_ESM.doc]

Additional file 1: **Courses of action in a suspected case of actual or potential child abuse in France**

Risk of abuse

where the physician notes deficient care or education

Suspected abuse

where the physician notes possible signs of abuse

Report

Report of "concern"

Judicial authorities Administrative authorities

To the county child abuse prevention office (*Cellule de recueil, de traitement et d’évaluation des informations préoccupantes,* CRIP)

To the **District Prosecutor** in writing or by phone with subsequent written confirmation (by fax and mail).

The physician must to keep a copy and make a copy to the county child abuse prevention office

The District Prosecutor begins a criminal investigation and also contacts the county social services.

The county child abuse prevention office starts to evaluate the child's situation. If necessary, the office she sends the file to the District Prosecutor with a view to legal proceedings
