# Supplementary material for: Risk factors for child abuse: levels of knowledge and difficulties in family medicine. A mixed method study
Source: BMC Res Notes. 2015 Oct 30;8:620. doi: 10.1186/s13104-015-1607-9 (PMC4627620; doi:10.1186/s13104-015-1607-9)
Supplement: Supplementary file 2 — 10.1186/s13104-015-1607-9 Risk factors for child abuse reported in the literature. [file 13104_2015_1607_MOESM2_ESM.doc]

Additional file 2: Risk factors for child abuse reported in the literature

| **Risk factors related to the child** | |
| --- | --- |
| In prenatal period | Unwanted pregnancy^19^ |
|  | Little or no monitoring during pregnancy^19, 20^ |
|  | Maternal hospitalization (due to a risk of premature delivery or to intrauterine growth restriction)^19^ |
| In the perinatal period | Premature < 37 weeks of amenorrhea^19, 26^ |
|  | Low birth weight < 5.5 lb^20^ |
| In the postnatal period | Young age^26, 18^ |
|  | Handicap (especially mental handicap) ^20^ |
|  | Behavioral disorders ^27^ |
|  | Several children in the family, and a history of abuse of the siblings^19, 20, 21^ |
| **Risk factors related to the parents** | |
| Pathological psychological problems ^17, 20^ | Psychiatric disorders |
|  | Depression |
|  | Post-partum depression |
| Drug and/or alcohol abuse^20, 22^ | |
| Parental history of abuse^19, 22^ | |
| Unstable family situation^5, 17, 19, 20, 26^ | Single mother |
|  | Blended family |
|  | Absence of a parent |
|  | Marital conflict and separation |
|  | Domestic violence and other family violence reported |
| Failure to provide parental care, especially by the mother^5, 19^ | |
| "Emotional deficiency" ^17^ | |
