# Supplementary material for: Risk factors for child abuse: levels of knowledge and difficulties in family medicine. A mixed method study
Source: BMC Res Notes. 2015 Oct 30;8:620. doi: 10.1186/s13104-015-1607-9 (PMC4627620; doi:10.1186/s13104-015-1607-9)
Supplement: Supplementary file 3 — 10.1186/s13104-015-1607-9 Questionnaire. [file 13104_2015_1607_MOESM3_ESM.doc]

Additional file 3: **QUESTIONNAIRE**

**INTERVIEW**

**Family physician and child abuse**

**Who is the Family physician?**

1. Name and last name :
2. Sex :  Male  Female
3. Year of birth:
4. When did he begin to work as a Family physician?
5. Year of the end of his/her residency :
6. Conditions of work :
   1. Town :
   2.  Rural areas  Semirural areas  Urban areas
   3.  Alone  with other MD  with other health professionals
   4. Do you work a part of your time in an hospital?

 Oui  Non

**About child abuse**

1. In your opinion, do risk factors of child abuse exist?

 Yes  No  Don’t know

1. In your FP’s experience, have you ever suspected a case (or more) of child abuse?

 Yes  No

**If YES at question 8, ask question 9 to 13**

1. If yes, what did you do?

………………………………………………………………………………………………………………………………………………………………………………………………………………………………………………………………………………………………………………………………………………………………

1. If you did something, was the child abuse confirmed?

 Yes  No  Don’t know

FP’s difficulties when child abuse is suspected

1. What were the difficulties encountered in cases of suspected child abuse?

…………………………………………………………………………………………………………………………………………………………………………………………………………………………………………………………………………………………………………………………………………………………………………………………………………………………………………………………………………………

1. Of the following statements, are they difficulties encountered in cases of suspected child abuse?
2. I fear of an incorrect diagnosis

 Strongly agree  Somewhat agree  Somewhat disagree  Strongly disagree

1. The reporting requires too much time.

 Strongly agree  Somewhat agree  Somewhat disagree  Strongly disagree

1. I have difficulties in controlling my emotions.

 Strongly agree  Somewhat agree  Somewhat disagree  Strongly disagree

1. I have difficulties in drafting my report.

 Strongly agree  Somewhat agree  Somewhat disagree  Strongly disagree

1. I have a breach in reporting procedure.

 Strongly agree  Somewhat agree  Somewhat disagree  Strongly disagree

1. I have a lack of awareness of professional resources.

 Strongly agree  Somewhat agree  Somewhat disagree  Strongly disagree

1. I feel alone.

 Strongly agree  Somewhat agree  Somewhat disagree  Strongly disagree

1. I fear of threats or reprisals.

 Strongly agree  Somewhat agree  Somewhat disagree  Strongly disagree

1. The family has a high socio-economics level.

 Strongly agree  Somewhat agree  Somewhat disagree  Strongly disagree

FP’s knowledge of child abuse risk factors

1. When you suspected child abuse, did identified risk factors of child abuse in the family? Yes No

If YES: 13a. Which ones?

…………………………………………………………………………...

…………………………………………………………………………...

…………………………………………………………………………...

13b. Finding this risk factors, did you more sure of your hypothesis?

 Yes  No

If NO: 13c. Now, do you think there were risk factors which could help you to be surer of your hypothesis?

 Yes  No

Can you explain? ……………………………………………………………………………………………………………………………………………………………………………………………………………………………......

**Go to question 16.**

**If NO at question 8, ask question 14 to 15.**

1. In case of child abuse suspicion, do you identify barriers in the support of this case?

 Yes  No

FP’s knowledge of child abuse risk factors

1. If you suspect a child abuse, do you think that the presence of risk factors could strengthen your diagnosis?

 Yes  No

………………………………………………………………………………………………………………………………………………………………………………………………………………………………………………………………………………

**For all FPs**

1. Regarding the following characteristics, do you consider them to be risk factors of child abuse?

Related to the child

1. Young age

 Strongly agree  Tend to agree  Tend to disagree  Strongly disagree

1. Prematurity

 Strongly agree  Tend to agree  Tend to disagree  Strongly disagree

1. Mental handicap

 Strongly agree  Tend to agree  Tend to disagree  Strongly disagree

1. Physical handicap

 Strongly agree  Tend to agree  Tend to disagree  Strongly disagree

1. Behavioural disorders

 Strongly agree  Tend to agree  Tend to disagree  Strongly disagree

Related to the parents

1. Emotional deficiency

 Strongly agree  Tend to agree  Tend to disagree  Strongly disagree

1. Failure to provide parental care

 Strongly agree  Tend to agree  Tend to disagree  Strongly disagree

1. Psychiatric disorders

 Strongly agree  Tend to agree  Tend to disagree  Strongly disagree

1. Depression (especially post-partum)

 Strongly agree  Tend to agree  Tend to disagree  Strongly disagree

1. Drug and/or alcohol abuse

 Strongly agree  Tend to agree  Tend to disagree  Strongly disagree

1. History of abuse in a parent

 Strongly agree  Tend to agree  Tend to disagree  Strongly disagree

1. Parental inability to meet the child's requirements

 Strongly agree  Tend to agree  Tend to disagree  Strongly disagree

1. Young maternal age

 Strongly agree  Tend to agree  Tend to disagree  Strongly disagree

1. Low maternal educational level

 Strongly agree  Tend to agree  Tend to disagree  Strongly disagree

1. Low socio-economic status

 Strongly agree  Tend to agree  Tend to disagree  Strongly disagree

1. If a quick and validated tool to screen child abuse or risk factors is available in French, could you use it?

.....................................................................................................................................................................................................................................................................................................................................................................................................................................................................

Physicians’ feelings about their role in caring for potential victims of child abuse

1. In your opinion, what are the FPs’ roles in caring for potential victims of child abuse?

………………………………………………………………………………………………………………………………………………………………………………………………………………………………………………………………………………..

1. Do you felt personally involved in caring for children at risk of child abuse?  Yes  No
2. What do you think about your involment in the process with the other stakeholders in the field of child abuse? ………………………………………………………………………………………………………………………………………………………………………………………………………………………………………………………………………

FPs training about child abuse

1. Did you receive training on detecting and managing child abuse? (Many answer authorized)

 Yes, during my medical education

 Yes, during my FPs practice

 No (Go to question 23)

1. (If Yes), was this training useful?

………………………………………………………………………………………………………………………………………………………………………………………………………………………………………………………………………………………

1. (If no), would you be interested to be trained on detecting and managing child abuse?

 Yes  No

1. What would be the modalities of training on chid abuse that perfectly meet your needs?

………………………………………………………………………………………………………………………………………………………………………………………………………………………………………………………………………

1. What would be useful to help you when you are suspect a child abuse situation?

………………………………………………………………………………………………………………………………………………………………………………………………………………………………………………………………………
